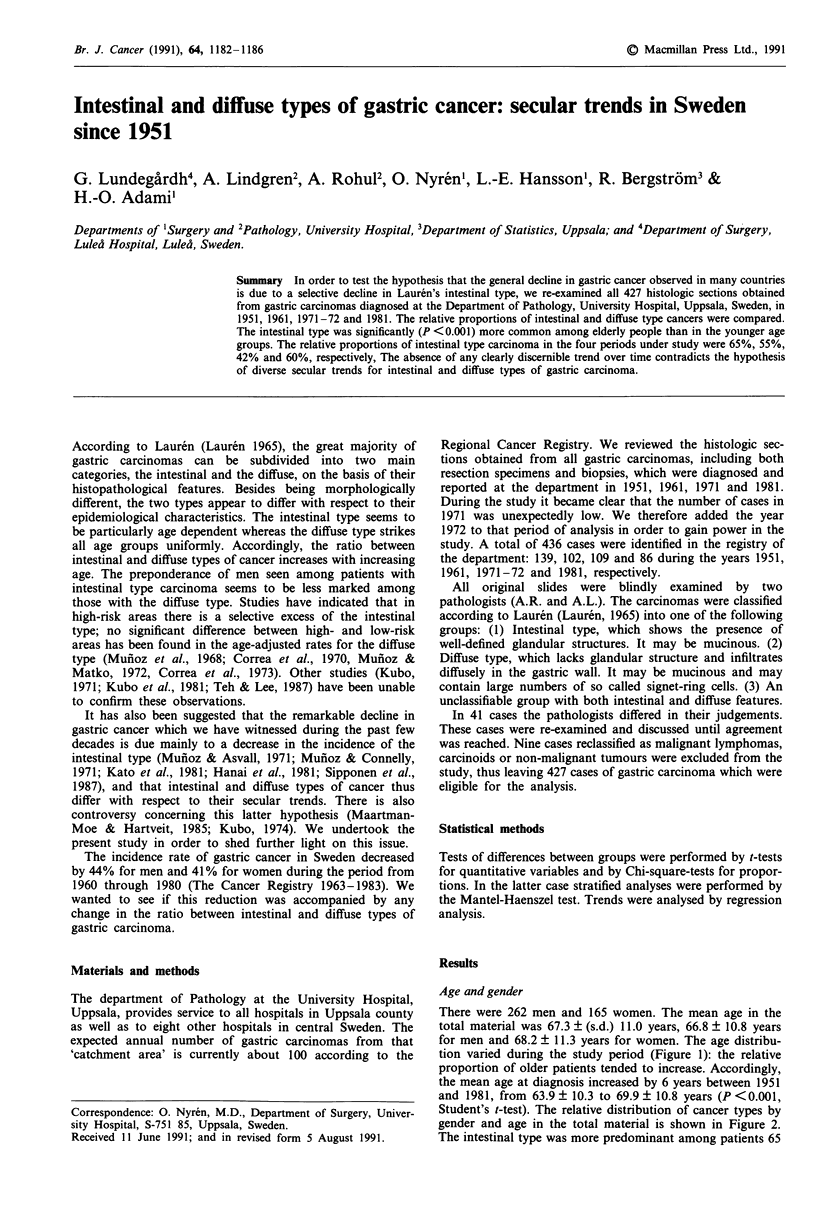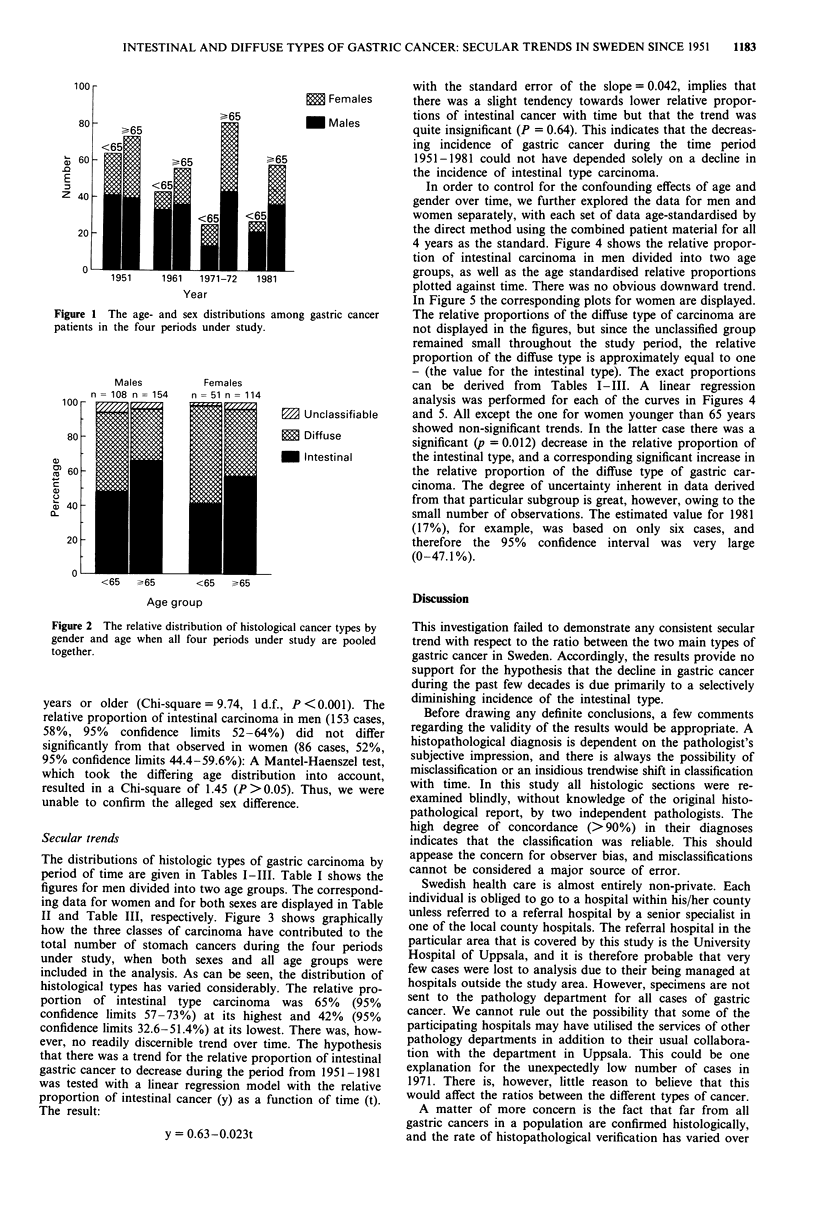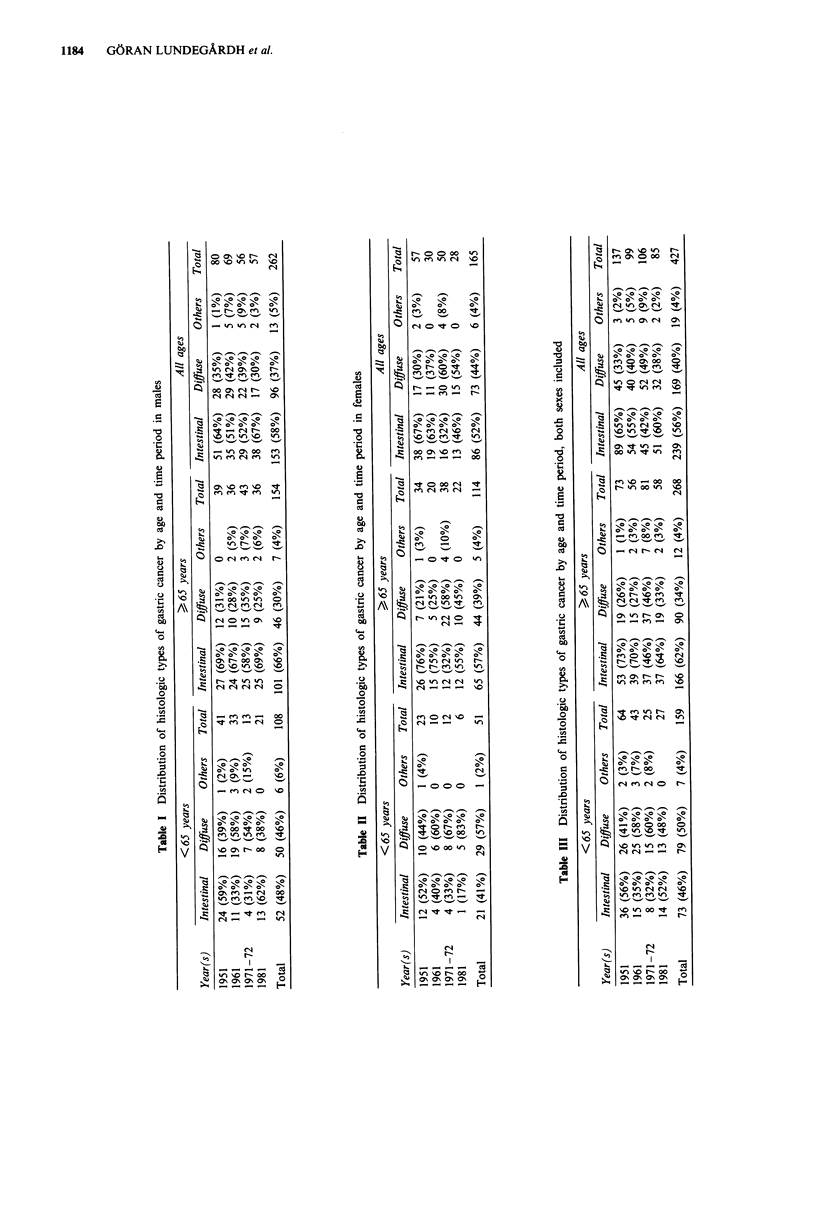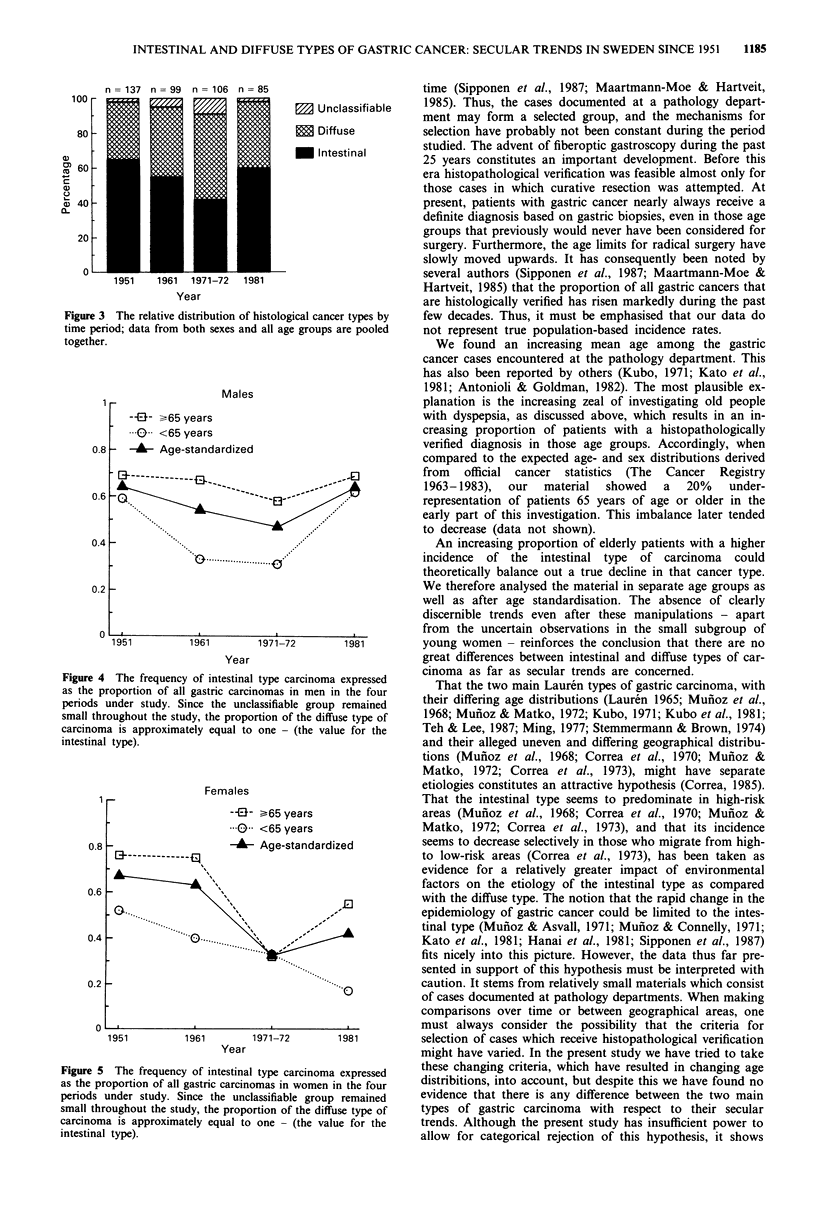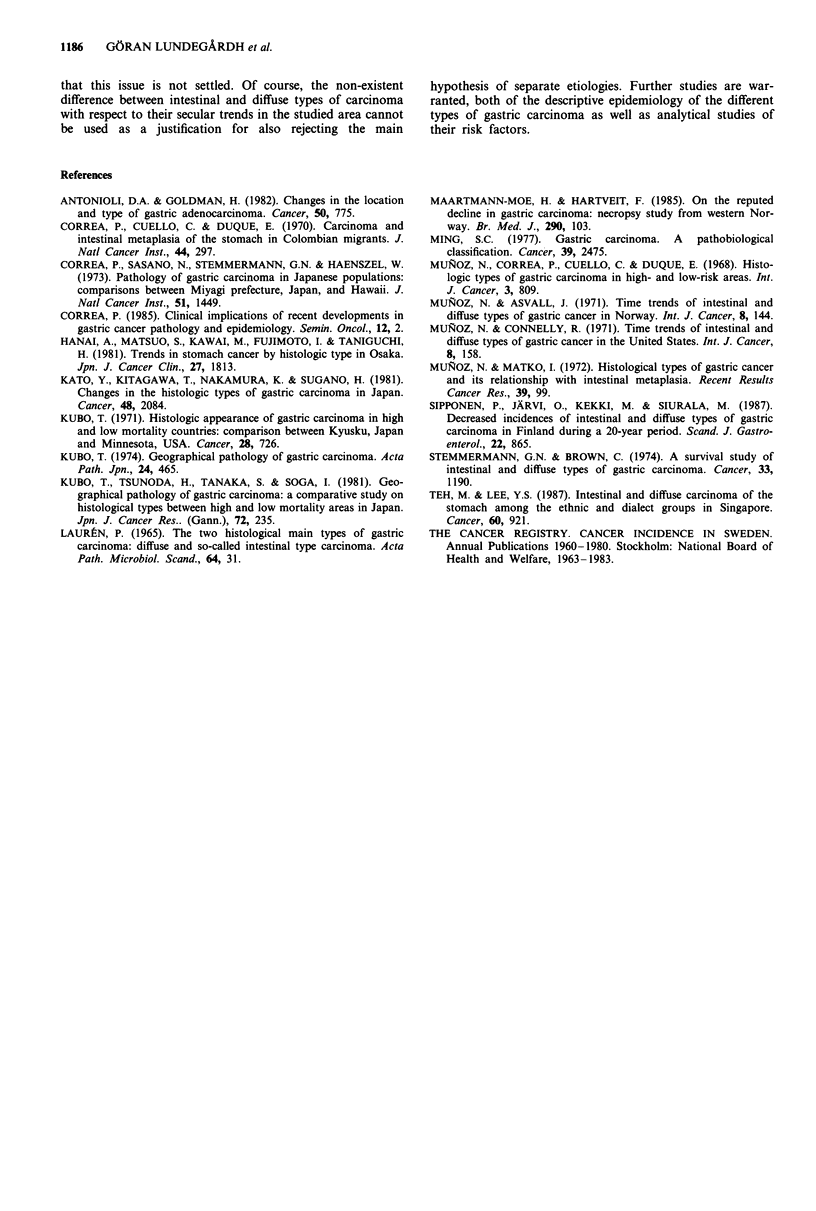# Intestinal and diffuse types of gastric cancer: secular trends in Sweden since 1951.

**DOI:** 10.1038/bjc.1991.487

**Published:** 1991-12

**Authors:** G. Lundegårdh, A. Lindgren, A. Rohul, O. Nyrén, L. E. Hansson, R. Bergström, H. O. Adami

**Affiliations:** Department of Surgery, University Hospital, Uppsala, Sweden.

## Abstract

In order to test the hypothesis that the general decline in gastric cancer observed in many countries is due to a selective decline in Laurén's intestinal type, we re-examined all 427 histologic sections obtained from gastric carcinomas diagnosed at the Department of Pathology, University Hospital, Uppsala, Sweden, in 1951, 1961, 1971-72 and 1981. The relative proportions of intestinal and diffuse type cancers were compared. The intestinal type was significantly (P less than 0.001) more common among elderly people than in the younger age groups. The relative proportions of intestinal type carcinoma in the four periods under study were 65%, 55%, 42% and 60%, respectively. The absence of any clearly discernible trend over time contradicts the hypothesis of diverse secular trends for intestinal and diffuse types of gastric carcinoma.


Br. J. Cancer (1991), 64, 1182–1186 © Macmillan Press Ltd., 1991

### Intestinal and diffuse types of gastric cancer: secular trends in Sweden since 1951

G. Lundegårdh[4], A. Lindgren[2], A. Rohul[2], O. Nyrén[1], L.-E. Hansson[1], R. Bergström[3] & H.-O. Adami[1]

*Departments of [1]Surgery and [2]Pathology, University Hospital, [3]Department of Statistics, Uppsala; and [4]Department of Surgery, Luleå Hospital, Luleå, Sweden.*

**Summary** In order to test the hypothesis that the general decline in gastric cancer observed in many countries is due to a selective decline in Laurén's intestinal type, we re-examined all 427 histologic sections obtained from gastric carcinomas diagnosed at the Department of Pathology, University Hospital, Uppsala, Sweden, in 1951, 1961, 1971–72 and 1981. The relative proportions of intestinal and diffuse type cancers were compared. The intestinal type was significantly ($P < 0.001$) more common among elderly people than in the younger age groups. The relative proportions of intestinal type carcinoma in the four periods under study were 65%, 55%, 42% and 60%, respectively, The absence of any clearly discernible trend over time contradicts the hypothesis of diverse secular trends for intestinal and diffuse types of gastric carcinoma.

According to Laurén (Laurén 1965), the great majority of gastric carcinomas can be subdivided into two main categories, the intestinal and the diffuse, on the basis of their histopathological features. Besides being morphologically different, the two types appear to differ with respect to their epidemiological characteristics. The intestinal type seems to be particularly age dependent whereas the diffuse type strikes all age groups uniformly. Accordingly, the ratio between intestinal and diffuse types of cancer increases with increasing age. The preponderance of men seen among patients with intestinal type carcinoma seems to be less marked among those with the diffuse type. Studies have indicated that in high-risk areas there is a selective excess of the intestinal type; no significant difference between high- and low-risk areas has been found in the age-adjusted rates for the diffuse type (Muñoz *et al.*, 1968; Correa *et al.*, 1970, Muñoz & Matko, 1972, Correa *et al.*, 1973). Other studies (Kubo, 1971; Kubo *et al.*, 1981; Teh & Lee, 1987) have been unable to confirm these observations.

It has also been suggested that the remarkable decline in gastric cancer which we have witnessed during the past few decades is due mainly to a decrease in the incidence of the intestinal type (Muñoz & Asvall, 1971; Muñoz & Connelly, 1971; Kato *et al.*, 1981; Hanai *et al.*, 1981; Sipponen *et al.*, 1987), and that intestinal and diffuse types of cancer thus differ with respect to their secular trends. There is also controversy concerning this latter hypothesis (Maartman-Moe & Hartveit, 1985; Kubo, 1974). We undertook the present study in order to shed further light on this issue.

The incidence rate of gastric cancer in Sweden decreased by 44% for men and 41% for women during the period from 1960 through 1980 (The Cancer Registry 1963–1983). We wanted to see if this reduction was accompanied by any change in the ratio between intestinal and diffuse types of gastric carcinoma.

### Materials and methods

The department of Pathology at the University Hospital, Uppsala, provides service to all hospitals in Uppsala county as well as to eight other hospitals in central Sweden. The expected annual number of gastric carcinomas from that 'catchment area' is currently about 100 according to the Regional Cancer Registry. We reviewed the histologic sections obtained from all gastric carcinomas, including both resection specimens and biopsies, which were diagnosed and reported at the department in 1951, 1961, 1971 and 1981. During the study it became clear that the number of cases in 1971 was unexpectedly low. We therefore added the year 1972 to that period of analysis in order to gain power in the study. A total of 436 cases were identified in the registry of the department: 139, 102, 109 and 86 during the years 1951, 1961, 1971–72 and 1981, respectively.

All original slides were blindly examined by two pathologists (A.R. and A.L.). The carcinomas were classified according to Laurén (Laurén, 1965) into one of the following groups: (1) Intestinal type, which shows the presence of well-defined glandular structures. It may be mucinous. (2) Diffuse type, which lacks glandular structure and infiltrates diffusely in the gastric wall. It may be mucinous and may contain large numbers of so called signet-ring cells. (3) An unclassifiable group with both intestinal and diffuse features.

In 41 cases the pathologists differed in their judgements. These cases were re-examined and discussed until agreement was reached. Nine cases reclassified as malignant lymphomas, carcinoids or non-malignant tumours were excluded from the study, thus leaving 427 cases of gastric carcinoma which were eligible for the analysis.

### Statistical methods

Tests of differences between groups were performed by *t*-tests for quantitative variables and by Chi-square-tests for proportions. In the latter case stratified analyses were performed by the Mantel-Haenszel test. Trends were analysed by regression analysis.

### Results

### *Age and gender*

There were 262 men and 165 women. The mean age in the total material was 67.3 ± (s.d.) 11.0 years, 66.8 ± 10.8 years for men and 68.2 ± 11.3 years for women. The age distribution varied during the study period (Figure 1): the relative proportion of older patients tended to increase. Accordingly, the mean age at diagnosis increased by 6 years between 1951 and 1981, from 63.9 ± 10.3 to 69.9 ± 10.8 years ($P < 0.001$, Student's *t*-test). The relative distribution of cancer types by gender and age in the total material is shown in Figure 2. The intestinal type was more predominant among patients 65

Correspondence: O. Nyrén, M.D., Department of Surgery, University Hospital, S-751 85, Uppsala, Sweden.
Received 11 June 1991; and in revised form 5 August 1991.

**Figure 1** The age- and sex distributions among gastric cancer patients in the four periods under study.

**Figure 2** The relative distribution of histological cancer types by gender and age when all four periods under study are pooled together.

years or older (Chi-square = 9.74, 1 d.f., $P < 0.001$). The relative proportion of intestinal carcinoma in men (153 cases, 58%, 95% confidence limits 52–64%) did not differ significantly from that observed in women (86 cases, 52%, 95% confidence limits 44.4–59.6%): A Mantel-Haenszel test, which took the differing age distribution into account, resulted in a Chi-square of 1.45 ($P > 0.05$). Thus, we were unable to confirm the alleged sex difference.

*Secular trends*

The distributions of histologic types of gastric carcinoma by period of time are given in Tables I–III. Table I shows the figures for men divided into two age groups. The corresponding data for women and for both sexes are displayed in Table II and Table III, respectively. Figure 3 shows graphically how the three classes of carcinoma have contributed to the total number of stomach cancers during the four periods under study, when both sexes and all age groups were included in the analysis. As can be seen, the distribution of histological types has varied considerably. The relative proportion of intestinal type carcinoma was 65% (95% confidence limits 57–73%) at its highest and 42% (95% confidence limits 32.6–51.4%) at its lowest. There was, however, no readily discernible trend over time. The hypothesis that there was a trend for the relative proportion of intestinal gastric cancer to decrease during the period from 1951–1981 was tested with a linear regression model with the relative proportion of intestinal cancer (y) as a function of time (t). The result:

$$y = 0.63 - 0.023t$$

with the standard error of the slope = 0.042, implies that there was a slight tendency towards lower relative proportions of intestinal cancer with time but that the trend was quite insignificant ($P = 0.64$). This indicates that the decreasing incidence of gastric cancer during the time period 1951–1981 could not have depended solely on a decline in the incidence of intestinal type carcinoma.

In order to control for the confounding effects of age and gender over time, we further explored the data for men and women separately, with each set of data age-standardised by the direct method using the combined patient material for all 4 years as the standard. Figure 4 shows the relative proportion of intestinal carcinoma in men divided into two age groups, as well as the age standardised relative proportions plotted against time. There was no obvious downward trend. In Figure 5 the corresponding plots for women are displayed. The relative proportions of the diffuse type of carcinoma are not displayed in the figures, but since the unclassified group remained small throughout the study period, the relative proportion of the diffuse type is approximately equal to one – (the value for the intestinal type). The exact proportions can be derived from Tables I–III. A linear regression analysis was performed for each of the curves in Figures 4 and 5. All except the one for women younger than 65 years showed non-significant trends. In the latter case there was a significant ($p = 0.012$) decrease in the relative proportion of the intestinal type, and a corresponding significant increase in the relative proportion of the diffuse type of gastric carcinoma. The degree of uncertainty inherent in data derived from that particular subgroup is great, however, owing to the small number of observations. The estimated value for 1981 (17%), for example, was based on only six cases, and therefore the 95% confidence interval was very large (0–47.1%).

### Discussion

This investigation failed to demonstrate any consistent secular trend with respect to the ratio between the two main types of gastric cancer in Sweden. Accordingly, the results provide no support for the hypothesis that the decline in gastric cancer during the past few decades is due primarily to a selectively diminishing incidence of the intestinal type.

Before drawing any definite conclusions, a few comments regarding the validity of the results would be appropriate. A histopathological diagnosis is dependent on the pathologist's subjective impression, and there is always the possibility of misclassification or an insidious trendwise shift in classification with time. In this study all histologic sections were reexamined blindly, without knowledge of the original histopathological report, by two independent pathologists. The high degree of concordance (>90%) in their diagnoses indicates that the classification was reliable. This should appease the concern for observer bias, and misclassifications cannot be considered a major source of error.

Swedish health care is almost entirely non-private. Each individual is obliged to go to a hospital within his/her county unless referred to a referral hospital by a senior specialist in one of the local county hospitals. The referral hospital in the particular area that is covered by this study is the University Hospital of Uppsala, and it is therefore probable that very few cases were lost to analysis due to their being managed at hospitals outside the study area. However, specimens are not sent to the pathology department for all cases of gastric cancer. We cannot rule out the possibility that some of the participating hospitals may have utilised the services of other pathology departments in addition to their usual collaboration with the department in Uppsala. This could be one explanation for the unexpectedly low number of cases in 1971. There is, however, little reason to believe that this would affect the ratios between the different types of cancer.

A matter of more concern is the fact that far from all gastric cancers in a population are confirmed histologically, and the rate of histopathological verification has varied over

**Table I** Distribution of histologic types of gastric cancer by age and time period in males

| | <65 years | | | | ≥65 years | | | | All ages | | | |
|---|---|---|---|---|---|---|---|---|---|---|---|---|
| *Year(s)* | *Intestinal* | *Diffuse* | *Others* | *Total* | *Intestinal* | *Diffuse* | *Others* | *Total* | *Intestinal* | *Diffuse* | *Others* | *Total* |
| 1951 | 24 (59%) | 16 (39%) | 1 (2%) | 41 | 27 (69%) | 12 (31%) | 0 | 39 | 51 (64%) | 28 (35%) | 1 (1%) | 80 |
| 1961 | 11 (33%) | 19 (58%) | 3 (9%) | 33 | 24 (67%) | 10 (28%) | 2 (5%) | 36 | 35 (51%) | 29 (42%) | 5 (7%) | 69 |
| 1971–72 | 4 (31%) | 7 (54%) | 2 (15%) | 13 | 25 (58%) | 15 (35%) | 3 (7%) | 43 | 29 (52%) | 22 (39%) | 5 (9%) | 56 |
| 1981 | 13 (62%) | 8 (38%) | 0 | 21 | 25 (69%) | 9 (25%) | 2 (6%) | 36 | 38 (67%) | 17 (30%) | 2 (3%) | 57 |
| Total | 52 (48%) | 50 (46%) | 6 (6%) | 108 | 101 (66%) | 46 (30%) | 7 (4%) | 154 | 153 (58%) | 96 (37%) | 13 (5%) | 262 |

**Table II** Distribution of histologic types of gastric cancer by age and time period in females

| | <65 years | | | | ≥65 years | | | | All ages | | | |
|---|---|---|---|---|---|---|---|---|---|---|---|---|
| *Year(s)* | *Intestinal* | *Diffuse* | *Others* | *Total* | *Intestinal* | *Diffuse* | *Others* | *Total* | *Intestinal* | *Diffuse* | *Others* | *Total* |
| 1951 | 12 (52%) | 10 (44%) | 1 (4%) | 23 | 26 (76%) | 7 (21%) | 1 (3%) | 34 | 38 (67%) | 17 (30%) | 2 (3%) | 57 |
| 1961 | 4 (40%) | 6 (60%) | 0 | 10 | 15 (75%) | 5 (25%) | 0 | 20 | 19 (63%) | 11 (37%) | 0 | 30 |
| 1971–72 | 4 (33%) | 8 (67%) | 0 | 12 | 12 (32%) | 22 (58%) | 4 (10%) | 38 | 16 (32%) | 30 (60%) | 4 (8%) | 50 |
| 1981 | 1 (17%) | 5 (83%) | 0 | 6 | 12 (55%) | 10 (45%) | 0 | 22 | 13 (46%) | 15 (54%) | 0 | 28 |
| Total | 21 (41%) | 29 (57%) | 1 (2%) | 51 | 65 (57%) | 44 (39%) | 5 (4%) | 114 | 86 (52%) | 73 (44%) | 6 (4%) | 165 |

**Table III** Distribution of histologic types of gastric cancer by age and time period, both sexes included

| | <65 years | | | | ≥65 years | | | | All ages | | | |
|---|---|---|---|---|---|---|---|---|---|---|---|---|
| *Year(s)* | *Intestinal* | *Diffuse* | *Others* | *Total* | *Intestinal* | *Diffuse* | *Others* | *Total* | *Intestinal* | *Diffuse* | *Others* | *Total* |
| 1951 | 36 (56%) | 26 (41%) | 2 (3%) | 64 | 53 (73%) | 19 (26%) | 1 (1%) | 73 | 89 (65%) | 45 (33%) | 3 (2%) | 137 |
| 1961 | 15 (35%) | 25 (58%) | 3 (7%) | 43 | 39 (70%) | 15 (27%) | 2 (3%) | 56 | 54 (55%) | 40 (40%) | 5 (5%) | 99 |
| 1971–72 | 8 (32%) | 15 (60%) | 2 (8%) | 25 | 37 (46%) | 37 (46%) | 7 (8%) | 81 | 45 (42%) | 52 (49%) | 9 (9%) | 106 |
| 1981 | 14 (52%) | 13 (48%) | 0 | 27 | 37 (64%) | 19 (33%) | 2 (3%) | 58 | 51 (60%) | 32 (38%) | 2 (2%) | 85 |
| Total | 73 (46%) | 79 (50%) | 7 (4%) | 159 | 166 (62%) | 90 (34%) | 12 (4%) | 268 | 239 (56%) | 169 (40%) | 19 (4%) | 427 |

**Figure 3** The relative distribution of histological cancer types by time period; data from both sexes and all age groups are pooled together.

**Figure 4** The frequency of intestinal type carcinoma expressed as the proportion of all gastric carcinomas in men in the four periods under study. Since the unclassifiable group remained small throughout the study, the proportion of the diffuse type of carcinoma is approximately equal to one – (the value for the intestinal type).

**Figure 5** The frequency of intestinal type carcinoma expressed as the proportion of all gastric carcinomas in women in the four periods under study. Since the unclassifiable group remained small throughout the study, the proportion of the diffuse type of carcinoma is approximately equal to one – (the value for the intestinal type).

time (Sipponen *et al.*, 1987; Maartmann-Moe & Hartveit, 1985). Thus, the cases documented at a pathology department may form a selected group, and the mechanisms for selection have probably not been constant during the period studied. The advent of fiberoptic gastroscopy during the past 25 years constitutes an important development. Before this era histopathological verification was feasible almost only for those cases in which curative resection was attempted. At present, patients with gastric cancer nearly always receive a definite diagnosis based on gastric biopsies, even in those age groups that previously would never have been considered for surgery. Furthermore, the age limits for radical surgery have slowly moved upwards. It has consequently been noted by several authors (Sipponen *et al.*, 1987; Maartmann-Moe & Hartveit, 1985) that the proportion of all gastric cancers that are histologically verified has risen markedly during the past few decades. Thus, it must be emphasised that our data do not represent true population-based incidence rates.

We found an increasing mean age among the gastric cancer cases encountered at the pathology department. This has also been reported by others (Kubo, 1971; Kato *et al.*, 1981; Antonioli & Goldman, 1982). The most plausible explanation is the increasing zeal of investigating old people with dyspepsia, as discussed above, which results in an increasing proportion of patients with a histopathologically verified diagnosis in those age groups. Accordingly, when compared to the expected age- and sex distributions derived from official cancer statistics (The Cancer Registry 1963–1983), our material showed a 20% under-representation of patients 65 years of age or older in the early part of this investigation. This imbalance later tended to decrease (data not shown).

An increasing proportion of elderly patients with a higher incidence of the intestinal type of carcinoma could theoretically balance out a true decline in that cancer type. We therefore analysed the material in separate age groups as well as after age standardisation. The absence of clearly discernible trends even after these manipulations – apart from the uncertain observations in the small subgroup of young women – reinforces the conclusion that there are no great differences between intestinal and diffuse types of carcinoma as far as secular trends are concerned.

That the two main Laurén types of gastric carcinoma, with their differing age distributions (Laurén 1965; Muñoz *et al.*, 1968; Muñoz & Matko, 1972; Kubo, 1971; Kubo *et al.*, 1981; Teh & Lee, 1987; Ming, 1977; Stemmermann & Brown, 1974) and their alleged uneven and differing geographical distributions (Muñoz *et al.*, 1968; Correa *et al.*, 1970; Muñoz & Matko, 1972; Correa *et al.*, 1973), might have separate etiologies constitutes an attractive hypothesis (Correa, 1985). That the intestinal type seems to predominate in high-risk areas (Muñoz *et al.*, 1968; Correa *et al.*, 1970; Muñoz & Matko, 1972; Correa *et al.*, 1973), and that its incidence seems to decrease selectively in those who migrate from high- to low-risk areas (Correa *et al.*, 1973), has been taken as evidence for a relatively greater impact of environmental factors on the etiology of the intestinal type as compared with the diffuse type. The notion that the rapid change in the epidemiology of gastric cancer could be limited to the intestinal type (Muñoz & Asvall, 1971; Muñoz & Connelly, 1971; Kato *et al.*, 1981; Hanai *et al.*, 1981; Sipponen *et al.*, 1987) fits nicely into this picture. However, the data thus far presented in support of this hypothesis must be interpreted with caution. It stems from relatively small materials which consist of cases documented at pathology departments. When making comparisons over time or between geographical areas, one must always consider the possibility that the criteria for selection of cases which receive histopathological verification might have varied. In the present study we have tried to take these changing criteria, which have resulted in changing age distribitions, into account, but despite this we have found no evidence that there is any difference between the two main types of gastric carcinoma with respect to their secular trends. Although the present study has insufficient power to allow for categorical rejection of this hypothesis, it shows that this issue is not settled. Of course, the non-existent difference between intestinal and diffuse types of carcinoma with respect to their secular trends in the studied area cannot be used as a justification for also rejecting the main hypothesis of separate etiologies. Further studies are warranted, both of the descriptive epidemiology of the different types of gastric carcinoma as well as analytical studies of their risk factors.

**References**

ANTONIOLI, D.A. & GOLDMAN, H. (1982). Changes in the location and type of gastric adenocarcinoma. *Cancer*, **50**, 775.

CORREA, P., CUELLO, C. & DUQUE, E. (1970). Carcinoma and intestinal metaplasia of the stomach in Colombian migrants. *J. Natl Cancer Inst.*, **44**, 297.

CORREA, P., SASANO, N., STEMMERMANN, G.N. & HAENSZEL, W. (1973). Pathology of gastric carcinoma in Japanese populations: comparisons between Miyagi prefecture, Japan, and Hawaii. *J. Natl Cancer Inst.*, **51**, 1449.

CORREA, P. (1985). Clinical implications of recent developments in gastric cancer pathology and epidemiology. *Semin. Oncol.*, **12**, 2.

HANAI, A., MATSUO, S., KAWAI, M., FUJIMOTO, I. & TANIGUCHI, H. (1981). Trends in stomach cancer by histologic type in Osaka. *Jpn. J. Cancer Clin.*, **27**, 1813.

KATO, Y., KITAGAWA, T., NAKAMURA, K. & SUGANO, H. (1981). Changes in the histologic types of gastric carcinoma in Japan. *Cancer*, **48**, 2084.

KUBO, T. (1971). Histologic appearance of gastric carcinoma in high and low mortality countries: comparison between Kyusku, Japan and Minnesota, USA. *Cancer*, **28**, 726.

KUBO, T. (1974). Geographical pathology of gastric carcinoma. *Acta Path. Jpn.*, **24**, 465.

KUBO, T., TSUNODA, H., TANAKA, S. & SOGA, I. (1981). Geographical pathology of gastric carcinoma: a comparative study on histological types between high and low mortality areas in Japan. *Jpn. J. Cancer Res.*. (Gann.), **72**, 235.

LAURÉN, P. (1965). The two histological main types of gastric carcinoma: diffuse and so-called intestinal type carcinoma. *Acta Path. Microbiol. Scand.*, **64**, 31.

MAARTMANN-MOE, H. & HARTVEIT, F. (1985). On the reputed decline in gastric carcinoma: necropsy study from western Norway. *Br. Med. J.*, **290**, 103.

MING, S.C. (1977). Gastric carcinoma. A pathobiological classification. *Cancer*, **39**, 2475.

MUÑOZ, N., CORREA, P., CUELLO, C. & DUQUE, E. (1968). Histologic types of gastric carcinoma in high- and low-risk areas. *Int. J. Cancer*, **3**, 809.

MUÑOZ, N. & ASVALL, J. (1971). Time trends of intestinal and diffuse types of gastric cancer in Norway. *Int. J. Cancer*, **8**, 144.

MUÑOZ, N. & CONNELLY, R. (1971). Time trends of intestinal and diffuse types of gastric cancer in the United States. *Int. J. Cancer*, **8**, 158.

MUÑOZ, N. & MATKO, I. (1972). Histological types of gastric cancer and its relationship with intestinal metaplasia. *Recent Results Cancer Res.*, **39**, 99.

SIPPONEN, P., JÄRVI, O., KEKKI, M. & SIURALA, M. (1987). Decreased incidences of intestinal and diffuse types of gastric carcinoma in Finland during a 20-year period. *Scand. J. Gastroenterol.*, **22**, 865.

STEMMERMANN, G.N. & BROWN, C. (1974). A survival study of intestinal and diffuse types of gastric carcinoma. *Cancer*, **33**, 1190.

TEH, M. & LEE, Y.S. (1987). Intestinal and diffuse carcinoma of the stomach among the ethnic and dialect groups in Singapore. *Cancer*, **60**, 921.

THE CANCER REGISTRY. CANCER INCIDENCE IN SWEDEN. Annual Publications 1960–1980. Stockholm: National Board of Health and Welfare, 1963–1983.